# Maxims for the Selection of Climate in Pulmunary, Laryngeal and Bone Tuberculosis

**Published:** 1907-02

**Authors:** 


### MAXIMS FOR THE SELECTION OF CLIMATE IN PULMONARY, LARYNGEAL, AND BONE TUBERCULOSIS.

---

Knopf contributes to the *New York Medical Journal* of July 28, 1906, some maxims of value. The value of any climate to a tuberculous patient cannot be determined or compared with any other unless the patient lives under careful medical guidance in a sanatorium or private home, and follows the best hygienic and dietetic treatment.

Laryngeal cases, in the earlier stages with relatively little pulmonary involvement, are often benefited by a change to a moderately high or to a warm, moist climate with between 500 and 1500 feet of altitude, particularly when there is a chronic feeling of dryness in the throat. When there is a tendency to moist catarrh, even the very dry and hot climates, provided dust storms are not frequent, are often beneficial. In pulmonary cases a weak heart, distinctive heart lesions, emphysema, constant fever, and extreme nervousness are contraindications to high altitudes. Whenever such pathological conditions as were just mentioned do not exist, great altitudes, and cold or cool or hot and dry regions, dust-free, or at least relatively dust-free, will always prove a valuable adjuvant in the treatment of tuberculosis. Early pulmonary hemorrhage *per se* need not be considered in determining a choice of climate. However, as a rule, and particularly in cases inclined to frequent hemoptyses, long distance journeys should be made with frequent rests and great altitudes approached only gradually. Not a few cases with slight bronchial symptoms (bronchitis), but weak hearts and evidences of mixed infection, do well at the seacoast for the greater part of the year. Prolonged ocean voyages may also prove beneficial in such cases. The patient, however, must not be subject to seasickness, must be fond of the ocean,and be in a situation to travel in comfort and with ease on a steamer or large ship, with a medical officer on board.

The ideal climate for the average pulmonary patient, in the eary and more hopeful stages of the disease, is the one where the extremes of temperature are not great, with the purest atmosphere, relatively little humidity, much sunshine, and all conditions which permit the patient to live comfortably out-of-doors the largest number of days out of the year, and the largest number of hours out of the twenty-four.

For tuberculosis of the bones and joints and scrofulous affections of childhood the seacoast climates in our temperate zones come nearer to deserving the term "specific" than anything else. The peculiarly beneficial influence of seacoast climate in bone and joint tuberculosis is doubtlessly to be ascribed to the aseptic and ozonic quality of the air, and iodides and other salts suspended therein. But pure air and outdoor life, in whatever locality, can and should be utilized in the treatment of tuberculosis and scrofulous affections of childhood.

In the choice of locality for the purpose of a climate change for any patient many factors must be taken into account. These are:

(a) If the patient wishes to return to his present home, after improvement or restoration to health, may he do so safely or not? Experience has shown that when great climatic changes have been made patients frequently relapse when returned to their former homes.

(*b*) If the patient is married or single, young or old, willing or unwilling to leave. If the patient is subject to nostalgia, and generally much attached to home environments, sending him far away, and particularly if against his wish, may often produce disastrous results, for mental depression retards recovery and aggravates pathological conditions.

(c) If the patient is sanguine and cheerful he will usually do well even at long distances from home, in isolated regions, within or outside of a sanitorium.

(d) For the morose and hypochondriac patient isolation or long distances away from home have often produced the same result as nostalgia.

(e) To remove an advanced and evidently hopeless case from his home to a long distance is as cruel as it is unscientific, unless it is done by the patient's special request and with the likelihood of obtaining the object in view, to lessen his sufferings and to make him happier in general. Slight climatic changes with short distances to travel are often beneficial in such far-advanced cases when made with the absolute consent of the patient.—*Thepentic Gazette,* Dec. 15, 1906.

